# Rethinking Celiac Disease Management: Treatment Approaches Beyond the Gluten-Free Diet

**DOI:** 10.3390/biomedicines14010029

**Published:** 2025-12-22

**Authors:** Dimitris Kounatidis, Argyro Pavlou, Apostolos Evangelopoulos, Maria Psaroudaki, Evangelia Kotsi, Ioanna Petrakou, Panagiotis Paraskevopoulos, Vasileios Stamatopoulos, Eleni Mylona, Natalia G. Vallianou

**Affiliations:** 1 Diabetes Center, First Propaedeutic Department of Internal Medicine, Medical School, National and Kapodistrian University of Athens, Laiko General Hospital, 11527 Athens, Greece; dimitriskounatidis82@outlook.com; 2 First Department of Internal Medicine, Sismanogleio General Hospital, 15126 Athens, Greece; argirpavlou@gmail.com (A.P.); marypsaroud@gmail.com (M.P.); panparask1@gmail.com (P.P.); 3 Medical School, National and Kapodistrian University of Athens, 11527 Athens, Greece; apostolos.evangelopoulos.nak@gmail.com (A.E.); joannapetrakou@hotmail.com (I.P.); 4 Second Department of Internal Medicine, Medical School, National and Kapodistrian University of Athens, Hippokration General Hospital, 11527 Athens, Greece; lila.kotsi@yahoo.com; 5 First Department of Internal Medicine, Evaggelismos General Hospital, 10676 Athens, Greece; kypseli96@gmail.com; 6 Fifth Department of Internal Medicine and Infectious Diseases, Evaggelismos General Hospital, 10676 Athens, Greece; mylonaelena@gmail.com

**Keywords:** celiac disease, gluten-free diet, interleukin-15, latiglutenase, machine learning, novel therapies, nutraceuticals, organ-on-chip systems, pathogenesis, transglutaminase, vaccination

## Abstract

Celiac disease (CeD) is a chronic, immune-mediated enteropathy triggered by dietary gluten in genetically susceptible individuals, with environmental and epigenetic factors also contributing to its pathogenesis. Once considered a rare pediatric malabsorptive disorder, CeD is now recognized as a systemic condition that can manifest with both gastrointestinal and extraintestinal symptoms across the lifespan. Although strict adherence to a gluten-free diet (GFD) remains the cornerstone of treatment, up to 30–40% of patients experience persistent symptoms and/or ongoing mucosal injury despite reported compliance. This therapeutic gap, combined with advances in molecular understanding of disease mechanisms, has driven the development of novel strategies targeting key pathogenic pathways. Intraluminal interventions include gluten-degrading enzymes and gluten-sequestering agents, while other approaches target tissue transglutaminase 2, induce antigen-specific immune tolerance, or modulate cytokine-driven inflammation, with particular emphasis on interleukin-15 (IL-15) signaling. Additional strategies aim to inhibit lymphocyte trafficking to the intestinal mucosa and enhance intestinal barrier function through zonulin modulation. Adjunctive therapies under investigation include nutraceuticals, microbiota-targeted interventions, and vaccine-based approaches. More recently, advanced experimental and computational platforms, such as human intestinal organoids, organ-on-chip systems, and machine learning–driven analytics, are being leveraged in efforts to accelerate translational research and support the rational design of precision medicine approaches. This narrative review synthesizes current evidence for therapies beyond the GFD, examines challenges in clinical implementation, and discusses how technological innovations may reshape the future therapeutic landscape of CeD.

## 1. Introduction

Celiac disease (CeD) is a chronic, immune-mediated enteropathy triggered by dietary gluten in genetically susceptible individuals, with a global prevalence estimated at 0.7–1.4% [1]. Once considered a rare pediatric malabsorptive disorder, CeD is now recognized as a systemic condition that can affect individuals throughout the lifespan. In addition to genetic predisposition, environmental and epigenetic factors are believed to contribute to disease onset by promoting a self-perpetuating inflammatory cascade, characterized by abnormal immune recognition of gluten-derived peptides. This cascade leads to progressive villous atrophy and crypt hyperplasia, resulting in impaired intestinal absorptive capacity and consequent maldigestion and malabsorption [2]. Clinically, CeD presents with a highly heterogeneous phenotype, encompassing a broad spectrum of both gastrointestinal and extraintestinal manifestations [3]. Diagnosis is typically established by detecting specific immunoglobulin A (IgA) antibodies against tissue transglutaminase 2 (TG2), followed by a small-intestinal biopsy. Despite increased awareness and advances in diagnostic methods, CeD remains substantially underdiagnosed, primarily due to its heterogeneous clinical presentations [4,5,6].

At present, lifelong adherence to a strict gluten-free diet (GFD) remains the only proven effective treatment. Most patients experience marked symptomatic improvement and gradual mucosal healing, with serological markers commonly declining within a period of 1–2 years. However, normalization of antibody titers does not always correlate with complete histological recovery [7]. Moreover, up to 30–40% of patients continue to report persistent symptoms and/or demonstrate ongoing mucosal inflammation despite self-reported adherence to the GFD [8]. This phenomenon is frequently attributed to inadvertent gluten exposure, most commonly through cross-contamination, as even trace levels of gluten below 50 mg/day may be sufficient to sustain mucosal damage [9]. The considerable practical limitations and psychosocial burden associated with long-term dietary restriction have therefore stimulated increasing interest in adjunctive and alternative therapeutic strategies aimed at improving disease control and quality of life (QoL) [10].

Emerging therapeutic approaches for CeD target multiple stages of disease pathogenesis, ranging from intraluminal neutralization of gluten to immune modulation and restoration of immune tolerance. Intraluminal strategies include oral gluten-sequestering compounds and gluten-degrading enzymes, which aim to prevent the generation and activity of immunogenic gluten peptides by binding or inactivating them prior to mucosal interaction. Downstream interventions, like tissue TG2 inhibitors, seek to attenuate antigen-driven immune activation by blocking gluten peptide deamidation. Tolerance-inducing strategies attempt to re-educate the immune system to recognize gluten antigens in a non-inflammatory context. Moreover, immune-modulatory therapies remain a major focus of investigation, including inhibitors of lymphocyte trafficking and monoclonal antibodies targeting pro-inflammatory cytokines, particularly interleukin (IL)-15) [11,12]. These approaches are of particular relevance in refractory celiac disease type II (RCD II), a clinical entity in which dietary treatment alone is frequently insufficient [12,13].

Increasing research activity has also focused on the potential beneficial effects of nutraceutical interventions, given that patients with CeD commonly exhibit deficiencies in micronutrients and vitamins. Growing evidence further implicates alterations in the gut microbiota in CeD pathogenesis. Consequently, therapeutic modulation of microbial composition and intestinal barrier function through tight junction regulators and biotic-based therapies is actively being explored [2,3]. In addition, novel vaccination platforms and technological innovations, including human intestinal organoids (HIOs), organ-on-chip (OOC) systems, and machine learning (ML)-based approaches, are creating new opportunities for improving therapeutic development and clinical outcomes [14,15,16].

This narrative review aims to synthesize recent advances in the development of novel therapeutic strategies for CeD. It first outlines key aspects of disease pathogenesis, clinical manifestations, diagnostic approaches, and principles of gluten-free dietary management. It then examines emerging nutraceutical, pharmacological, immunological, microbiome-based, and vaccine-oriented interventions, and highlights the current gaps that limit translational progress and clinical implementation. Particular emphasis is placed on the role of technological innovations in shaping future therapeutic paradigms. Ultimately, this review seeks to provide clinicians and researchers with a comprehensive overview of the rapidly evolving therapeutic landscape in CeD, extending beyond conventional dietary management.

## 2. Pathogenesis

### 2.1. Main Risk Factors

#### 2.1.1. Genetic Factors

A prerequisite for the development of CeD is genetic susceptibility. However, currently identified genetic loci account for only approximately 50% of the disease heritability, with the majority of inherited risk mapping to the human leukocyte antigen (HLA) region. The principal genetic determinants are the HLA-DQA1 and HLA-DQB1 genes, which encode the HLA-DQ2 and HLA-DQ8 heterodimers and are present in nearly all affected individuals. These molecules are essential for the presentation of gluten-derived peptides to CD4^+^ T lymphocytes and are indispensable for the initiation of the pathogenic immune response [17]. Beyond the HLA region, genome-wide association studies (GWAS) have identified more than 100 non-HLA susceptibility loci that contribute to CeD risk [18,19]. More recently, an increased burden of genetic variants within the *BTN2A1* locus has been reported, with multiple single-nucleotide polymorphisms (SNPs) within *BTN2A1*, *BTN3A1*, and *BTN3A2* demonstrating significant associations with CeD. Notably, these correlations appear largely independent of HLA risk haplotypes [20].

#### 2.1.2. Environmental Factors

A range of environmental factors has been proposed to influence the development of CeD, including gastrointestinal infections, early and/or repeated exposure to antibiotics, infant feeding practices, and both the timing and amount of gluten introduced during infancy [21]. Several viral pathogens, such as rotavirus, enterovirus, and adenovirus, have been implicated as potential triggers of disease onset [22]. About two decades ago, a prospective study provided early evidence that recurrent gastrointestinal rotavirus infections may increase the risk of CeD in genetically susceptible children [23]. Observational data further suggest that a higher frequency of respiratory infections during the first two years of life may distinguish children who later develop CeD from those who remain unaffected [24]. On the other hand, although numerous studies showed that early-life nutritional exposures can promote a transition from oral tolerance to pathogenic immune responses to gluten, more recent randomized controlled trials have challenged this association [21]. Interest has also grown in the potential impact of air pollutants on CeD development, with evidence reporting a genetic causal link [25]. Persistent organic pollutants (POPs), particularly p,p′-dichlorodiphenyldichloroethylene (DDE), have been associated with disease onset, with higher serum DDE levels being correlated with a two-fold increased risk of CeD [26]. Air pollutants may also exert effects in part through alterations in gut microbiota, although direct evidence is lacking [27]. Overall, the relationship between air pollutants and CeD is still in the early stages of investigation and warrants further exploration in future large-scale studies.

#### 2.1.3. Epigenetic Factors

Epigenetic mechanisms, including DNA methylation, histone modifications, and non-coding RNAs such as microRNAs, appear to influence disease expression [28]. Recent research supports that loss of DNA methylation is a reversible epigenetic hallmark of CeD, occurring alongside the immune response in patients on a strict GFD. In particular, hypomethylation of long interspersed nuclear elements 1 (LINE-1) may facilitate cellular adaptation during gliadin-driven inflammation [29]. Furthermore, long non-coding RNAs (lncRNAs) have emerged as important modulators in CeD pathogenesis. For example, RP4-587D13.2 has been implicated in tight junction dysfunction, while lnc13 has been associated with CeD through its interaction with histone acetylase 1, forming a complex that regulates inflammatory gene expression. Additionally, upregulation of human endogenous retroviruses (HERVs) and dysregulation of the tripartite motif containing 28/SET domain bifurcated histone lysine methyltransferase 1 (TRIM28/SETDB1) complex have been reported as contributing factors in disease pathogenesis [30,31].

In summary, the interplay among genetic, environmental, and epigenetic influences appears to establish a permissive landscape for CeD emergence. Although the precise contributions of these factors remain incompletely defined and, at times, contentious, substantial advances have been achieved in recent years. Continued elucidation of these mechanisms will be essential for enhancing patient management and informing the development of novel therapeutic strategies.

### 2.2. Main Pathogenetic Mechanisms

#### 2.2.1. The Core Immunological Cascade

The pathogenesis of CeD is driven by a dysregulated immune response to gluten, a protein enriched in glutamine- and proline-containing sequences that impede complete enzymatic degradation [32]. Partially digested gluten peptides that traverse the intestinal epithelium and enter the lamina propria are subsequently modified by tissue TG2, which, upon calcium-dependent activation, catalyzes their deamidation and markedly enhances their immunogenicity [33]. The resulting deamidated epitopes bind with high affinity to HLA-DQ2 or HLA-DQ8 molecules on antigen-presenting cells (APCs), establishing a highly selective immunological interface that triggers the activation of gluten-specific CD4^+^ T cells. This interaction reprograms APCs toward a pro-inflammatory phenotype characterized by strong IL-15 secretion, which reshapes the epithelial compartment by conferring heightened cytotoxicity to CD8^+^ intraepithelial lymphocytes (IELs) and natural killer (NK) cells, while concurrently undermining regulatory T (Treg) cell–mediated immune restraint. Activated CD4^+^ T cells also drive a B cell response uniquely tailored to the CeD microenvironment, resulting in the production of high-affinity antibodies against both gluten antigens and TG2. In parallel, CD4^+^ T cell–derived interferon-gamma (IFN-γ) and IL-21 synergize with IL-15 to further potentiate IEL cytotoxicity and sustain epithelial stress. The cumulative effect of these signals, including IL-15–mediated production of tumor necrosis factor-alpha (TNF-α) and activation of the Fas (CD95)/Fas ligand (FasL) apoptotic pathway in enterocytes, establishes a chronic, self-perpetuating inflammatory milieu. The resulting sustained immune activation progressively devastates the small-intestinal mucosa, ultimately producing villous atrophy, crypt hyperplasia, and impaired nutrient absorption [32,33,34,35,36,37].

#### 2.2.2. The Role of the Gut Microbiota

In recent years, the intestinal microbiota has gained prominence as a key determinant in the pathogenesis of CeD. Gut dysbiosis, an imbalance in the composition and metabolic activity of commensal microorganisms, promotes mucosal immune disruption and weakens epithelial barrier function [38]. In CeD, this microbial shift typically involves an overrepresentation of Gram-negative taxa such as *Bacteroides*, *Proteobacteria*, and *Escherichia coli*, accompanied by reduced levels of beneficial Gram-positive genera including *Bifidobacterium* and *Lactobacillus*. Such alterations undermine tight junction integrity and increase paracellular permeability, facilitating the translocation of gluten peptides and microbial products into the lamina propria. This enhanced permeability establishes a “leaky gut” phenotype that intensifies mucosal immune activation [39,40]. Tight junctions consist of more than 40 structural and regulatory molecules, with occludin, claudins, and junctional adhesion molecules (JAMs) forming the core transmembrane components that preserve epithelial cohesion [41,42]. Zonulin, the only identified physiological modulator of tight junctions in humans, is elevated in contexts of heightened permeability and impaired junctional function [43]. Elevated luminal lipopolysaccharide (LPS) levels derived from Gram-negative bacteria activate innate immune pathways via toll-like receptors (TLR2 and TLR4), perpetuating cytokine-driven inflammation [44]. The microbiota also influences both gluten immunogenicity and immune tolerance. Certain bacterial proteases detoxify gluten peptides, whereas others, such as those secreted by *Pseudomonas aeruginosa*, generate more immunogenic fragments [45]. A balanced microbiome supports Treg differentiation and anti-inflammatory cytokine production through short-chain fatty acids (SCFAs), particularly butyrate. In contrast, CeD-associated dysbiosis reduces butyrate levels, impairs forkhead box P3 (FOXP3) expression, and lowers the threshold for gluten-specific T cell activation [46].

## 3. Clinical Presentation and Diagnostic Evaluation

### 3.1. Main Clinical Manifestations

The most common gastrointestinal manifestation of CeD is chronic diarrhea with steatorrhea, often accompanied by abdominal discomfort, distension, bloating, and excessive flatulence. These symptoms commonly contribute to weight loss and, in more advanced cases, overt protein–calorie malnutrition. Malabsorption of micronutrients and trace elements underlies many of the extraintestinal features. Iron deficiency anemia, frequently compounded by concomitant vitamin B12 or folate deficiency, and metabolic bone disease, including osteopenia and osteoporosis, are among the most prevalent complications. Less commonly, patients may present with reproductive, neurological, or psychiatric disturbances. A distinct cutaneous manifestation, dermatitis herpetiformis, may also occur. This condition is characterized by intensely pruritic vesiculopapular eruptions symmetrically distributed on extensor surfaces such as the elbows, knees, and buttocks. Notably, extraintestinal symptoms may predominate and, in some individuals, may arise even in the absence of overt gastrointestinal complaints [47,48,49].

### 3.2. Diagnostic Principles and Approach

**Figure 1:**
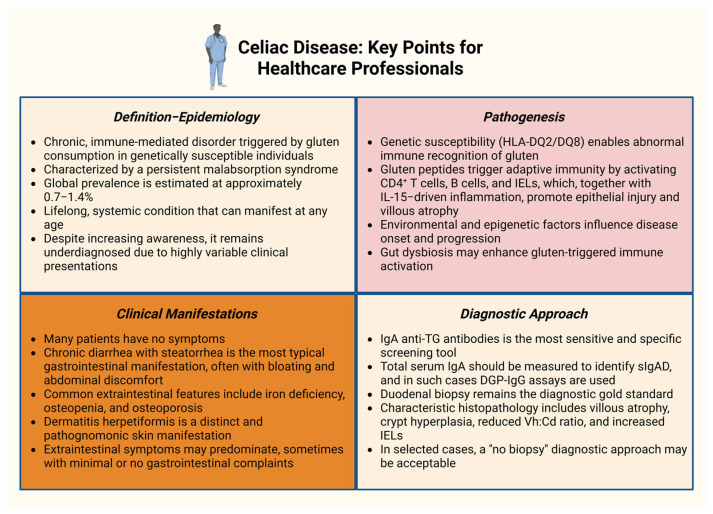
Celiac disease: Key points for healthcare professionals [1,2,4,17,21,28,32,39,47,48,49,50,51,52,53,54,57]. Abbreviations: DGP: deamidated gliadin peptides; HLA: human leukocyte antigen; IEL: intraepithelial lymphocyte; IgA: immunoglobulin A; IgG: immunoglobulin G; IL-15: interleukin-15; sIgAD: selective IgA deficiency; TG2: transglutaminase 2; Vh:Cd ratio: villous height to crypt depth ratio. Created in BioRender. Kounatidis, D. (2025) https://BioRender.com/na11rud (assessed on 18 December 2025).

The preferred initial diagnostic strategy for CeD is serological testing, with serum IgA anti-TG antibodies serving as the most sensitive and specific screening tool, exhibiting a diagnostic sensitivity up to 90–98%. Because selective IgA deficiency (sIgAD) occurs more frequently in CeD than in the general population, concurrent measurement of total serum IgA is essential to prevent false-negative results. In patients with sIgAD, immunoglobulin G (IgG)-based assays, particularly those directed against deamidated gliadin peptides (DGP-IgG), provide a reliable alternative [50,51,52]. While positive serology strongly supports diagnosis, histological confirmation through duodenal biopsy remains the diagnostic gold standard [53]. Current guidelines recommend obtaining at least four specimens from the distal duodenum and two from the duodenal bulb to maximize diagnostic yield [54]. Characteristic histopathological features of CeD include villous atrophy, crypt hyperplasia, a reduced villus height to crypt depth (Vh:Cd) ratio, and an increased abundance of IELs [55]. Endoscopic assessment remains essential for long-term monitoring, as the disease carries an elevated risk of malignancies, including enteropathy-associated T-cell lymphoma (EATL), particularly among patients with RCDII [56]. Of note, in selected cases, a “no biopsy” diagnostic approach is acceptable, particularly in individuals with biopsy-confirmed dermatitis herpetiformis or markedly elevated IgA anti-TG titers (≥10× the upper limit of normal) [54,57]. Figure 1 highlights the key characteristics of CeD.

## 4. The Gluten-Free Diet

The cornerstone of CeD management and the only established effective therapy at present is strict lifelong adherence to a GFD. This approach requires the complete exclusion of proteins derived from wheat, barley, and rye [58]. Although compliance with a GFD usually results in significant symptom relief and mucosal healing in most patients, it remains far from an ideal therapeutic approach because it is associated with substantial practical, nutritional, and psychosocial challenges. Achieving a completely gluten-free lifestyle is inherently difficult since even trace amounts of gluten are sufficient to trigger an immune-mediated response and cause intestinal damage [59].

In a prospective, double-blind, placebo-controlled study, Catassi et al., demonstrated that the continuous consumption of only 50 mg of gluten per day, which is approximately equivalent to a single breadcrumb and commonly encountered through cross-contamination, was enough to induce histological deterioration in nearly half of the CeD patients examined [60]. The problem of inadvertent exposure was further highlighted in a large-scale study based on crowd-sourced data from restaurant meals. Gluten contamination was detected in about 32% of dishes labeled as “gluten-free,” with pizza and pasta being the most frequently affected foods [61]. These findings illustrate the considerable difficulties faced by individuals with CeD in maintaining strict gluten avoidance. Further challenges include the high financial cost of gluten-free products, the risk of nutritional deficiencies such as insufficient fiber, iron, calcium, and B vitamins, and the significant emotional and social strain associated with long-term dietary restriction [62].

## 5. Potential Benefits with Nutraceuticals

In recent years, growing interest has focused on whether nutraceutical compounds could serve as helpful add-on therapies in the management of CeD. This interest largely stems from their reported anti-inflammatory effects and their potential to support the intestinal barrier. Preclinical studies, for example, suggest that curcumin may reduce gliadin-induced epithelial damage by dampening inflammatory pathways and helping maintain normal epithelial cell architecture [63]. Other polyphenols, such as resveratrol and quercetin may also show promise because they combine anti-inflammatory and antioxidant effects with broader immunomodulatory actions. Resveratrol, for instance, appears to influence immune signaling in the gut microenvironment and may help lessen intestinal symptoms [64]. On the other hand, quercetin may help regulate T helper cells and Tregs and has also been shown to shift the composition of the gut microbiota. In particular, it can decrease CeD-associated species like *Clostridium celatum* and *Bacteroides acidifaciens*, while increasing beneficial bacteria such as *Butyricicoccus pullicaecorum* and *Bifidobacterium longum*, changes that together help reduce gluten-triggered intestinal inflammation [65]. Micronutrients have also been explored for their therapeutic potential. Zinc supplementation, for example, has been linked to improvements in intestinal barrier structure and function, including reduced permeability and improved mucosal integrity [66]. Vitamin D has also received particular attention due to its role in immune regulation and its importance for maintaining a healthy epithelial barrier. However, evidence supporting its use in CeD remains unclear due to small and heterogeneous studies [67].

Overall, nutraceuticals have demonstrated promising effects across several pathways implicated in the pathophysiology of CeD. Their potential benefits may also extend to the epigenetic mechanisms of the disease, as similar actions have been reported in a range of other human disorders. Nevertheless, despite these encouraging observations, to date, no nutraceutical has been integrated into routine clinical management of CeD. This is primarily because much of the available data are derived from preclinical data, while the clinical studies conducted so far remain limited by small sample sizes, methodological limitations, and insufficient evidence regarding their long-term efficacy and safety. Another major challenge is that many nutraceuticals have low solubility and poor oral bioavailability, partly due to their extensive first-pass metabolism in the liver [68]. Interestingly, recent research showed that nutraceuticals may have mixed effects on inflammation, simultaneously reducing nuclear factor kappa-light-chain-enhancer of activated B cells (NF-κB) activity while increasing IL-6 and IL-10 expression, highlighting the complexity of their biological actions [69].

## 6. Novel Therapeutic Approaches Beyond Gluten Restriction

The incomplete efficacy of the GFD, together with advances in the understanding of CeD pathogenesis, has prompted the development of adjunctive therapeutic strategies intended to complement dietary treatment and improve patient-reported outcomes. Current investigational approaches focus on selectively modulating key molecular pathways involved in disease initiation and perpetuation. Orally administered endopeptidases are designed to degrade immunogenic gluten-derived peptides within the gastrointestinal lumen, thereby reducing the antigenic load before epithelial translocation and subsequent immune activation [70]. Other strategies aim to prevent luminal gluten–mucosal interactions through peptide sequestration or neutralization, limiting the generation of highly immunostimulatory fragments. TG2 has emerged as a particularly attractive therapeutic target owing to its vital role in deamidating glutamine residues within gluten peptides, a modification that increases their binding affinity for HLA-DQ2/DQ8 molecules and facilitates the activation of gluten-specific CD4^+^ T cells. Pharmacologic TG2 inhibition appears to attenuate downstream inflammatory cascades, responsible for villous atrophy and crypt hyperplasia [71].

Tolerization-based therapies pursue the restoration of antigen-specific immune tolerance by dampening maladaptive T-cell responses [72], while lymphocyte trafficking inhibitors aim to reduce the recruitment of effector lymphocytes to the intestinal mucosa, thereby mitigating local inflammation [73]. Increasing interest has also focused on IL-15 immunomodulation, given the central role of this cytokine in driving the cytotoxic activity of IELs and its critical involvement in the pathogenesis and persistence of refractory CeD [74]. Finally, therapeutic efforts have expanded toward barrier-directed interventions, with zonulin inhibitors representing a prominent class. This emphasis reflects the pivotal function of zonulin in regulating tight junction integrity; inhibiting its activity may improve intestinal permeability and limit the translocation of immunogenic peptides [43]. The following subsections describe the principal therapeutic agents currently under development that target these mechanisms.

### 6.1. Gluten Degradation

The enzymatic detoxification of gliadin peptides represents a promising adjunctive strategy for the management of CeD. Latiglutenase, also known as ALV003, is among the most extensively investigated candidates and consists of two recombinant proteases, ALV001 (a cysteine endoprotease B isoform) and ALV002 (a prolyl endopeptidase). This mixture remains stable under fed gastric conditions and efficiently hydrolyzes immunogenic gluten peptides into non-toxic fragments, thereby reducing their capacity to activate pathogenic CD4^+^ T cells. Single oral doses have been well tolerated, with no reports of serious adverse reactions [75,76].

Several studies have evaluated the clinical efficacy of latiglutenase in CeD, although the results have been heterogeneous. Evidence indicates that its therapeutic effects may be confined to seropositive patients, with no measurable benefits in seronegative individuals. Over a three-month treatment period, latiglutenase produced a dose-dependent and statistically significant reduction in disease-related symptoms, with the greatest improvements observed at the 900 mg dose. These benefits were accompanied by gains in QoL, particularly among participants with more severe baseline symptoms [77]. In a phase 2 trial, the administration of 1200 mg latiglutenase to patients undergoing a daily 2 g gluten challenge for six weeks attenuated gluten-induced mucosal injury and symptom severity. Compared with placebo, treatment was associated with a smaller reduction in the Vh:Cd ratio (−0.04 vs. −0.35; *p* = 0.057) and a lower increase in IEL density (9.8 vs. 24.8 cells/mm epithelium; *p* = 0.018) [78]. Similar histological outcomes were reported in another six-week gluten-challenge study using the same 2 g daily dose, although clinical symptoms did not differ significantly between groups [79]. In contrast, other clinical data indicate that latiglutenase did not improve either histologic or symptom scores compared with placebo [80]. The variability of outcomes across these trials is further reflected in a recent meta-analysis, which concluded that latiglutenase did not significantly influence histological endpoints relative to placebo [81].

Beyond latiglutenase, additional evidence has emerged for *Aspergillus niger* prolyl endopeptidase (AN-PEP), a proteolytic enzyme capable of degrading gluten within the stomach by cleaving the proline-rich peptide bonds characteristic of gluten proteins, thereby limiting the amount of intact gluten that reaches the small intestine [82]. Its potential as an oral therapeutic agent is supported by notable enzymatic stability across a broad acidic pH range (2–8), which confers resistance to gastric pepsin digestion. However, its proteolytic efficiency may be modulated by dietary components, as interactions with other food proteins may diminish its gluten-degrading ability. Beyond clinical applications, AN-PEP has been utilized in industrial biotechnology, particularly in the production of gluten-reduced or gluten-free foods through recombinant *A. niger* strains and in brewing processes [83]. Experimental data demonstrated that flour treated with AN-PEP does not elicit inflammatory or immune activation in cellular or animal models, distinguishing it from conventional wheat flour [84]. Clinical investigations in patients with CeD confirmed a favorable safety and tolerability profile, with no evidence of clinical, serological, or histological deterioration [85]. Nevertheless, its therapeutic efficacy remains uncertain. In a recent exploratory double-blind, randomized, placebo-controlled trial conducted in CeD patients adhering to long-term GFD, AN-PEP did not significantly reduce stool gluten immunogenic peptide (GIP) concentrations or alter CeD-specific serological markers, although it was associated with a lower proportion of participants reporting severe gastrointestinal symptoms [86].

Limited evidence is also available for the computationally designed endopeptidase TAK-062, a novel enzyme engineered for enhanced gluten degradation. In the sole phase 1 trial reported to date, TAK-062 achieved rapid in vitro degradation of more than 99% of gluten within ten minutes and demonstrated excellent tolerability in human subjects [87]. A recently completed clinical trial (NCT05353985) further evaluated TAK-062 in CeD patients undergoing gluten challenge, assessing safety, gastrointestinal symptoms, and mucosal outcomes. However, the formal results of this study have not yet been published.

### 6.2. Gluten Sequestration

The principal compound in this category is BL-7010, a high–molecular-weight, non-absorbable polymer previously designated as P(HEMA-co-SS). This polymer binds directly to gliadin within the intestinal lumen, shielding it from enzymatic degradation by digestive proteases and thereby preventing the formation of immunogenic gluten-derived peptides [72]. Evidence supporting the mechanism of BL-7010 derives primarily from preclinical studies. In vitro analyses have demonstrated its strong binding affinity for gliadin, while in vivo models have shown that BL-7010 effectively prevents gluten-induced mucosal damage. These protective effects include normalization of the Vh:Cd ratio, reduction in intraepithelial lymphocytosis, and preservation of epithelial barrier integrity, as evidenced by stable paracellular permeability and unaltered anion transporter-1 mRNA expression in the small intestine. Toxicological assessments in rats confirmed favorable tolerability and safety following repeated oral administration at doses up to 3000 mg/kg for 14 days, with no mutagenic activity observed in genetic toxicity assays [88]. At the clinical front, results are awaited from a randomized, double-blind, placebo-controlled trial (NCT01990885) designed to evaluate the safety of BL-7010 under both single and repeated dosing conditions in patients with well-controlled CeD, as well as to assess potential systemic absorption.

Promising findings have also emerged from an alternative gluten-sequestering strategy utilizing an orally administered egg yolk–derived anti-gliadin antibody, namely AGY. This investigational agent is specifically designed to neutralize GIP and thereby augment the protective effects of a GFD. In an open-label, single-arm pilot study involving ten adults with biopsy-confirmed CeD who had adhered to a strict GFD for at least six months, AGY showed a favorable safety and tolerability profile. Participants showed notable improvements in gastrointestinal symptoms, serological antibody titers reductions, and enhancements in QoL measures [89]. Nevertheless, the current evidence base remains limited to this preliminary study. Further validation is anticipated from a randomized, double-blind, placebo-controlled, crossover trial (NCT03707730) enrolling 149 individuals with biopsy-proven CeD maintained on a GFD, which aims to more comprehensively evaluate the safety and therapeutic efficacy of AGY.

### 6.3. Transglutaminase 2 Inhibition

The selective small-molecule TG2 inhibitor ZED1227 stands out as the leading therapeutic candidate in this class. Following oral administration, ZED1227 preferentially accumulates within villous enterocytes of the duodenum, where it covalently binds to the active form of TG2 through interaction with its catalytic cysteine residue. By blocking TG2 activity at this site, the inhibitor prevents the deamidation of gliadin peptides and their subsequent translocation into the lamina propria, thereby interrupting the cascade of immune activation [90,91].

A recent transcriptomic analysis of duodenal biopsies from individuals with CeD has provided further evidence supporting the efficacy of ZED1227. In participants receiving 100 mg of ZED1227 daily during a six-week gluten challenge, the intestinal mucosa was effectively protected from gluten-induced injury and inflammation. Gene expression profiles associated with mucosal architecture, epithelial differentiation, and nutrient absorption were largely preserved, remaining comparable to those observed in individuals adhering to a long-term GFD. Approximately half of the gluten-induced transcriptional changes were linked to epithelial IFN-γ responses, suggesting that modulation of epithelial immune signaling contributes substantially to the protective mechanism of ZED1227. Moreover, stratification based on HLA-DQ2 or HLA-DQ8 genotype may further enhance the mucosal protective effect of the inhibitor [92]. The first clinical evaluation of ZED1227 as a therapeutic agent for CeD was conducted in a phase 2, randomized, placebo-controlled proof-of-concept trial. Adults with well-controlled CeD underwent daily gluten challenge for six weeks while receiving oral doses of 10 mg, 50 mg, or 100 mg of ZED1227, or placebo. Across all treatment groups, ZED1227 significantly reduced gluten-induced mucosal damage, as evidenced by improved Vh:Cd ratios and reduced IEL counts, with the most pronounced benefits observed at higher doses. Adverse events were generally mild, most commonly headache, nausea, and diarrhea, and no major safety concerns were identified [93].

Additionally, a single-center, randomized, open-label study (NCT05818956) recently evaluated a related compound, TAK-227. This investigation examined the effects of food intake on the pharmacokinetics of the compound in healthy volunteers and assessed its tolerability and plasma concentration profiles to determine optimal dosing parameters. The study has been completed, and publication of its findings is awaited.

### 6.4. Gluten Tolerization

Among these approaches, TAK-101 has emerged as one of the most promising agents. This formulation comprises gliadin encapsulated within negatively charged poly(dl-lactide-co-glycolic acid) nanoparticles, specifically engineered to induce gluten-specific immune tolerance. Clinical evaluation through a phase 1 dose-escalation study and a phase 2a randomized, double-blind, placebo-controlled trial demonstrated both safety and biological activity. In the phase 2a study, individuals with well-controlled CeD underwent a 14-day gluten challenge. Treatment with TAK-101 resulted in an 88% reduction in gliadin-specific IFN-γ–producing cells relative to placebo, reflecting a marked suppression of immune activation. Whereas participants in the placebo group exhibited histological deterioration, those receiving TAK-101 largely maintained normal mucosal architecture. The therapy also led to decreased frequencies of circulating effector memory T cells expressing gut-homing markers, including α4β7^+^CD4^+^, αEβ7^+^CD8^+^, and γδ T cells, indicating attenuation of intestinal inflammatory pathways. Across all tested doses up to 8 mg/kg, TAK-101 was well tolerated, with no serious adverse events or clinically significant laboratory abnormalities [94].

A comparable immunological approach is being explored with KAN-101, a deamidated gliadin peptide conjugated to a liver-targeting glycosylation motif that directs the compound to hepatic tissues, thereby leveraging the liver’s inherent tolerogenic properties. In a phase 1 trial involving adults with biopsy-confirmed CeD carrying the HLA-DQ2.5 genotype, the agent’s safety, pharmacokinetics, and preliminary immunologic effects were assessed. During the single ascending dose phase, participants received intravenous KAN-101 at doses up to 1.5 mg/kg. Reported treatment-related adverse events were mild to moderate, including transient nausea, diarrhea, abdominal discomfort, and vomiting, with no serious or dose-limiting toxicities. Pharmacokinetic analysis indicated rapid systemic clearance, with dose-dependent half-lives ranging from approximately 4 to 30 min and complete elimination within six hours. In the multiple ascending dose phase, participants received three administrations of KAN-101 or placebo followed by a controlled gluten challenge. Adverse events remained mild, transient, and non-cumulative, with no evidence of drug accumulation or delayed toxicity. Collectively, KAN-101 exhibited consistent pharmacokinetic behavior, good tolerability, and a predictable safety profile across all tested dose levels [95].

### 6.5. Lymphocyte Migration Inhibition

Teriflunomide, an inhibitor of de novo pyrimidine synthesis that limits the proliferation and migration of both T and B cells, has been explored as a potential therapeutic option for CeD. Owing to its immunomodulatory properties, teriflunomide has been approved by the U.S. Food and Drug Administration (FDA) for the treatment of multiple sclerosis since 2012 [96]. In a phase 2a study involving 15 patients with CeD, Risnes et al. reported no significant differences in T cell activation markers between individuals treated with teriflunomide and those receiving placebo following gluten exposure. Based on these findings, the investigators concluded that teriflunomide is unlikely to represent an effective therapeutic strategy for CeD [97]. Notably, isolated case reports have linked the use of teriflunomide to small intestinal inflammation resembling CeD, as well as to lymphocytic colitis [98,99].

Beyond teriflunomide, two additional agents targeting lymphocyte migration are currently under active clinical investigation. The first, CCX282-B, is an oral antagonist of the CC chemokine receptor 9 (CCR9). Together with its ligand, the CC chemokine ligand 25 (CCL25), CCR9 forms a signaling axis that mediates the recruitment of inflammatory cells from secondary lymphoid tissues to the small intestine and is implicated in the pathogenesis of CeD. A phase 2 trial (NCT00540657) is evaluating CCX282-B at a dose of 250 mg twice daily in adults with CeD, comparing changes in the Vh:Cd ratio before and after gluten exposure relative to placebo. Although results have not yet been published, the study registry was most recently updated in 2025. Another compound, PTG-100, is an inhibitor of the α4β7 integrin, which mediates intestinal homing of effector T cells through interaction with the mucosal addressin cell adhesion molecule 1 (MAdCAM-1). PTG-100 has been evaluated in a phase 1 trial (NCT04524221) at a dose of 600 mg twice daily to assess safety, tolerability, and preliminary efficacy in CeD, with results pending.

### 6.6. Interleukin-15 Inhibition

Particular attention has focused on the janus kinase (JAK) inhibitor tofacitinib, an agent currently approved for the treatment of other autoimmune disorders, including rheumatoid arthritis and ulcerative colitis [100]. Preclinical data have demonstrated tofacitinib’s potential efficacy in experimental models of CeD. In an IL-15–transgenic mouse model, the drug led to a rapid decline in circulating NK cells within 10 days, followed by progressive depletion of pathogenic CD8^+^ T cells expressing high levels of the natural killer group 2D (NKG2D) receptor. This finding is clinically relevant, as NKG2D signaling delivers co-stimulatory activation signals that enhance cytotoxic T cell–mediated epithelial injury. Upon treatment completion, peripheral blood T cell profiles normalized, and CD8^+^NKG2D^+^ cell frequencies in the small intestinal mucosa were markedly reduced, reflecting effective suppression of the pathogenic immune cascade [101,102]. The central role of IL-15 has also been validated ex vivo. In intestinal mucosal explants derived from untreated CeD patients, IL-15 neutralization with a monoclonal antibody reduced enterocyte apoptosis, limited the release of cytotoxic effector molecules such as perforin, and suppressed IFN-γ secretion, thereby attenuating excessive mucosal immune activation [36].

Despite this robust preclinical rationale, the clinical translation of IL-15–targeted therapies in CeD has proven challenging. In a small pilot study involving patients with refractory CeD, tofacitinib produced rapid symptomatic relief and histological improvement; however, predefined immunologic endpoints were not achieved, and disease relapse occurred upon treatment cessation [103]. Similarly, the IL-15–neutralizing monoclonal antibody AMG 714 yielded modest symptomatic benefits but failed to induce significant changes in IEL density or villous architecture in phase 2a clinical trials [104,105]. The results of two recent phase 2 clinical trials are eagerly awaited. These studies evaluated the efficacy and safety of AMG 714 in adult patients with RCDII (NCT02633020), and the safety of three dosing regimens of the novel agent PRV-015 in adults with non-responsive CeD who continue to experience symptoms despite strict adherence to a GFD (NCT04424927).

### 6.7. Intestinal Barrier Modulation

Among pharmacological strategies aimed at restoring barrier integrity, larazotide acetate (AT-1001) has been the most extensively investigated. This synthetic octapeptide functions as a zonulin inhibitor and is administered orally. Its proposed mechanism involves preventing tight junction disassembly, thereby limiting gliadin translocation and dampening downstream immune activation [106]. In an initial clinical trial involving 20 patients challenged with 2.5 g of gluten, daily administration of 12 mg of larazotide acetate for seven days significantly reduced intestinal permeability, as evidenced by a decreased lactulose-to-mannitol (LAMA) ratio. The treatment also resulted in lower serum IFN-γ concentrations and improvements in gastrointestinal symptom scores [107].

Subsequent clinical trials confirmed the safety and tolerability of larazotide acetate, though efficacy outcomes have been variable. In a clinical trial of 86 participants with CeD, subjects were randomized to receive larazotide acetate at four different doses (0.25 mg, 1 mg, 4 mg, or 8 mg) or placebo, administered three times daily, with or without a gluten challenge of 2.4 g/day for a period of 2 weeks. The study showed that larazotide acetate improved gluten-induced gastrointestinal symptoms at some of the lower doses, whereas higher doses did not demonstrate consistent benefits. Assessment of intestinal permeability via the LAMA ratio was limited due to variability in the outpatient setting, preventing precise evaluation of the drug’s impact on barrier function [108]. A later phase 2 trial involving 184 patients demonstrated a reduction in anti-TG IgA concentrations with larazotide acetate doses ranging from 1 mg to 8 mg, although changes in intestinal permeability remained statistically non-significant [109]. A larger phase 2b study of 342 patients with persistent symptoms despite adherence to a GFD found that the 1.5 mg dose of larazotide acetate significantly improved gastrointestinal symptom scores, whereas higher doses (3 mg and 6 mg) yielded mixed results [110]. Notably, the phase 3 clinical trial (NCT03569007), which was designed to evaluate the efficacy and safety of larazotide acetate in alleviating persistent symptoms in adults with CeD adhering to a GFD, was prematurely discontinued by the sponsor.

**Figure 2:**
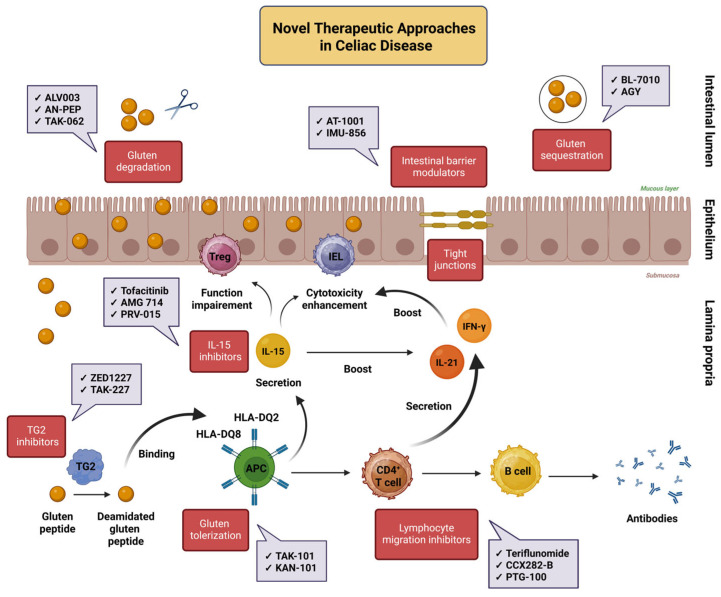
Novel therapeutic approaches in celiac disease. This figure illustrates the key molecular and immunological targets of novel pharmacological therapies for CeD. Intraluminal interventions reduce the availability of immunogenic gluten peptides through enzymatic degradation or sequestration, preventing epithelial injury. TG2 inhibitors act at the level of peptide deamidation to limit antigen-specific T cell activation, while tolerization agents aim to re-educate gluten-specific immune responses. Modulation of intestinal inflammation may also be achieved through the use of lymphocyte trafficking inhibitors, which seek to mitigate the recruitment of effector lymphocytes to the intestinal mucosa, as well as IL-15 inhibitors. The latter target the central activity of IL-15, which mediates deleterious effects by enhancing CD8^+^ IEL cytotoxicity, suppressing Treg function, and promoting IL-21 and IFN-γ production by CD4^+^ T cells. The field also includes intestinal barrier modulators, which act either by inhibiting zonulin, a key regulator of tight junction function, or by targeting epigenetic mechanisms that promote intestinal epithelial regeneration [43,70,71,72,73,74]. Abbreviations: APC: antigen-presenting cell; CeD: celiac disease; HLA: human leukocyte antigen; IEL: intraepithelial lymphocyte; IL: interleukin; IFN-γ: interferon-gamma; TG2: transglutaminase 2; Treg: regulatory T cell. Created in BioRender. Kounatidis, D. (2025) https://BioRender.com/07tpocy (assessed on 18 December 2025).

More recently, research interest has turned to the therapeutic potential of epigenetic regulators in CeD. One such agent is IMU-856, an oral, small-molecule modulator of sirtuin-6 (SIRT6), a protein that functions as a transcriptional regulator involved in the regeneration of the intestinal epithelium. Results from a phase 1 trial indicated a favorable safety profile and preliminary evidence of biological activity in patients with CeD [111,112]. Figure 2 illustrates the principal pharmacological interventions currently under investigation for CeD, emphasizing their primary targets within the disease’s pathogenic cascade. Subsequently, Table 1 and Table 2 present main findings from major completed and ongoing clinical studies evaluating these therapeutic approaches.

## 7. Microbiota-Targeted Therapies: The Potential Role of Biotic-Based Agents

Over the past decade, modulation of the gut microbiota has emerged as a promising therapeutic strategy for restoring microbial homeostasis and reducing disease activity in CeD. Several microbiota-directed interventions have been examined, with probiotics, prebiotics, and synbiotics being assessed in both preclinical and clinical settings [72,113].

Probiotics, characterized as live microorganisms that confer health benefits when administered in adequate amounts, have been the focus of increasing interest in CeD research [114]. Preclinical data demonstrated that *Bifidobacterium* spp. possess anti-inflammatory properties, reflected in an increased cyclooxygenase-1/cyclooxygenase-2 (COX-1/COX-2) ratio and suppression of major pro-inflammatory mediators such as IL-1β, TNF-α, and IFN-γ [115]. These strains may also facilitate degradation of immunogenic gliadin peptides and contribute to the induction of mucosal immune tolerance. *Bacillus amyloliquefaciens* EG025, isolated from traditional Korean fermented soybean paste, similarly exhibits gliadin-degrading capacity, strong acid and bile resistance, and a genomic profile indicating functional stability and safety, including the absence of antibiotic resistance genes or virulence determinants [116]. Clinical evidence supports the adjunctive use of probiotics alongside a GFD. In a randomized controlled trial, a multi-strain probiotic formulation containing *Lactobacillus* spp., *Bacillus* spp., and bacterial proteases significantly lowered residual fecal gluten relative to placebo and altered gut microbial composition and fecal metabolites associated with immune regulation, particularly SCFAs and branched-chain amino acid (BCAA) derivatives [117]. In another trial, 90-day supplementation with *Bifidobacterium lactis* CCT 7858 and *Lactobacillus rhamnosus* CCT 7863 improved gastrointestinal symptoms, emotional well-being, and overall QoL in patients with CeD [118].

Prebiotics, defined as non-digestible dietary substrates that selectively stimulate the growth and activity of beneficial gut microbes, have also been evaluated in CeD [119]. In vitro data from Brazil showed that cassava (*Manihot esculenta*) substrates promote the proliferation of *Lacticaseibacillus casei*, *Lactobacillus acidophilus*, and *Bifidobacterium animalis*, while shifting the microbial community toward beneficial taxa [120]. Human trials remain limited; however, a 12-week placebo-controlled study reported that combining a GFD with oligofructose-enriched inulin (10 g/day) increased epithelial surface area and reduced fecal calprotectin and serum antigen translocation, findings consistent with improved intestinal barrier integrity [121]. Synbiotics, which integrate probiotics with complementary prebiotic substrates, represent an additional strategy for optimizing gut ecological balance. Evidence indicates that pseudocereal-based synbiotic formulations reduce potentially deleterious taxa, like *Ruminococcaceae*, *Lachnospiraceae*, *Helicobacteraceae*, *Clostridium*, and *Escherichia*, while enriching beneficial genera, such as *Peptoclostridium*, *Prevotellaceae*, *Lactobacillus*, *Bifidobacterium*, *Enterococcus*, and *Eubacteriaceae* [122].

Notably, a recent hypothesis has proposed a role for probiotics in modulating the gut–skin axis, with potential therapeutic benefits for dermatitis herpetiformis [123]. Additional benefits may also arise from fecal microbiota transplantation (FMT), which has been successfully applied in several other autoimmune disorders, including inflammatory bowel disease. However, current evidence in CeD is extremely limited and is confined to a single case report involving refractory CeD with recurrent *Clostridioides difficile* infection, in which FMT restored duodenal villous architecture and alleviated gastrointestinal symptoms [124]. Intriguingly, the emerging intersection between the gut microbiome and epigenomic reprogramming represents a particularly promising area of research, given the central role of epigenetic mechanisms in CeD pathogenesis [125].

Overall, gut microbiota modulation constitutes a highly promising therapeutic approach for CeD. Nevertheless, it is essential to underscore that much of the existing evidence originates from experimental studies, while clinical research remains in its early stages. Considering the chronic and multifactorial nature of CeD, more comprehensive and long-term clinical trials are necessary to determine the sustained efficacy and safety of these biotic-based interventions.

## 8. The Role of Vaccination

### 8.1. Routine Immunization in Patients with Celiac Disease

Although formal vaccination guidelines tailored specifically to CeD are lacking, current clinical practice supports adherence to national to age-appropriate national immunization programs irrespective of the timing of diagnosis. Among commonly administered vaccines, rotavirus immunization has attracted particular scientific interest, given that, as previously discussed, rotavirus infection has been implicated as a potential environmental trigger in the pathogenesis of CeD. Early-life rotavirus vaccination appears to exert a protective effect by limiting infection-associated intestinal inflammation and preserving oral tolerance to gluten [126]. Population-based evidence corroborate this association, reporting significantly lower rates of CeD in vaccinated children and adolescents, with risk reduction persisting for at least 4 to 6 years post-immunization [126,127,128].

### 8.2. Development of Disease-Specific Vaccines: The Case of Nexvax2

Nexvax2, the first vaccine specifically targeting celiac disease, was developed approximately fifteen years ago. It contains three synthetic gluten peptides designed to induce immune tolerance by selectively modulating T cell reactivity. The vaccine targets individuals carrying HLA-DQ2, particularly those with the HLA-DQ2.5 haplotype, with the aim of reducing intestinal inflammation, supporting mucosal recovery, and ultimately allowing gluten exposure without triggering an immune response. Its mechanism of action resembles that of allergen desensitization therapies [129].

The first study to investigate the vaccine was a multicenter trial conducted in the United States and Oceania, assessing intradermal Nexvax2 in HLA-DQ2.5–positive adults with CeD who were maintained on a strict GFD. The trial demonstrated dose-dependent reductions in IFN-γ responses to vaccine peptides, without histologic deterioration of duodenal mucosa. Adverse events, including nausea, vomiting, headache, were predominantly mild to moderate, and the maximum tolerated dose was defined as 150 μg, as higher doses elicited transient gastrointestinal symptoms comparable to gluten exposure [130]. On the other hand, a randomized, double-blind, placebo-controlled trial conducted in the same year implemented a gradual dose-escalation protocol (3–900 μg), which prevented the cytokine release and gastrointestinal reactions previously observed with higher initial doses. Duodenal biopsies suggested a trend toward histologic improvement, indicating that larger doses could be administered safely if titrated progressively [131].

Subcutaneous administration yielded comparable immunologic outcomes, achieving similar peptide bioavailability without increasing IL-2 levels [132]. However, a later phase 2 trial using subcutaneous Nexvax2 (up to 900 μg twice weekly for 16 weeks) did not demonstrate superiority over placebo in mitigating gluten-induced gastrointestinal symptoms. Nonetheless, the study confirmed the feasibility of a short, blinded gluten challenge protocol, providing an essential methodological platform for future CeD immunotherapy research [133]. Interestingly, a more recent phase 2 study (RESET CeD) incorporated an improved cytokine release assay (CRA) to quantify gluten-specific CD4^+^ T cell activity without prior gluten challenge. The whole-blood CRA demonstrated greater sensitivity than PBMC-based assays, enabling accurate quantification of IL-2 and IFN-γ release. Nexvax2 administration at a 900 μg maintenance dose resulted in significant reductions in cytokine release and T cell proliferation compared with placebo, supporting the use of whole-blood CRA as a sensitive biomarker in CeD immunotherapy trials [134]. Despite these advances, Nexvax2 has not achieved sufficient clinical efficacy for therapeutic application.

### 8.3. Emerging Concepts in Celiac Disease Vaccination

**Figure 3:**
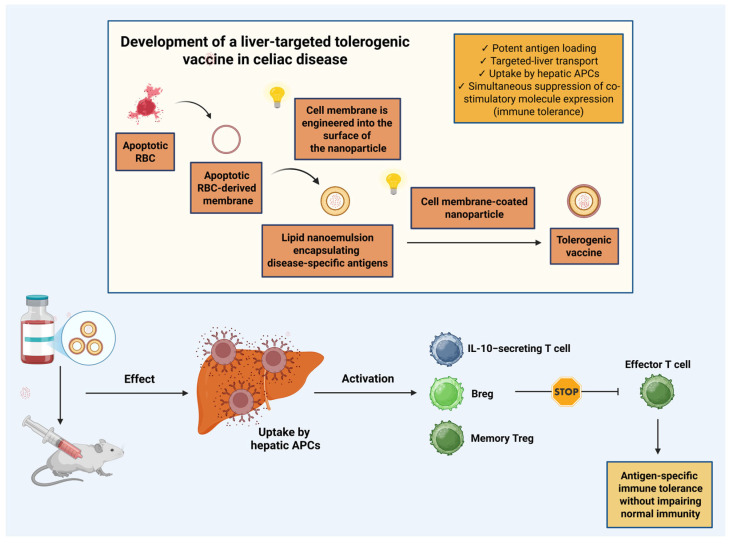
Development of a liver-targeted tolerogenic vaccine in celiac disease. This figure illustrates the key features and the development process of a nanoemulsion-based tolerogenic vaccine platform, as well as its mechanism of inducing antigen-specific immune tolerance without compromising normal immune function [137]. Abbreviations: APC: antigen-presenting cell; Breg: regulatory B cell; IL-10: interleukin-10; RBC: red blood cell; Treg: regulatory T cell. Created in BioRender. Kounatidis, D. (2025) https://BioRender.com/fi6kztl (assessed on 18 December 2025).

Beyond conventional and CeD-specific immunization strategies, increasing research interest has focused on inverse vaccination, a concept proposing restoration of immune tolerance through antigen presentation in a non-inflammatory context. Unlike traditional vaccines that induce protective immunity, inverse vaccines aim to activate regulatory pathways that suppress pathogenic responses to disease-relevant antigens [135,136]. A recent tolerogenic platform exemplifies this strategy by delivering selected antigens to hepatic APCs using lipid nanoemulsions incorporating membranes from apoptotic erythrocytes. This configuration allows efficient antigen loading, targeted hepatic delivery, and minimal systemic immune activation. In preclinical models, intravenous administration triggered multiple tolerogenic pathways, including induction of IL-10–producing T cells, regulatory B cells, and long-lived memory regulatory T-cell subsets. These effects promoted durable antigen-specific immune tolerance while maintaining overall immunocompetence [137]. The formulation process, principal characteristics, and immunological consequences of administering this groundbreaking vaccine are depicted in Figure 3.

## 9. Limitations, Challenges, and Future Perspectives

### 9.1. Limitations and Challenges

Although research on emerging therapeutic approaches for CeD has progressed significantly, no agent has achieved regulatory approval as an adjunct to the GFD [3,72]. This lack of approval reflects central methodological limitations, including substantial heterogeneity in study design, the enrollment of relatively small patient cohorts, and a predominant reliance on short-term efficacy and safety outcomes [73,138]. Notably, the gluten challenge model, widely used to provoke symptom recurrence, is increasingly viewed as suboptimal, due to a pronounced nocebo effect where participants frequently report symptoms even under placebo exposure [94,104]. A ‘’trial effect’’ has also been observed, as both treatment and placebo groups often exhibit improvements, likely reflecting enhanced dietary compliance and the psychological context of study participation [65]. Even when underlying pathogenic mechanisms are well-defined, targeted therapeutic approaches may fail to produce meaningful clinical outcomes. For instance, teriflunomide has not demonstrated the anticipated therapeutic benefits, and its use has been associated with the development of colitis, which could be particularly detrimental for patients with CeD [97,98,99].

Traditional histological endpoints, such as the Vh:Cd ratio, remain unvalidated as surrogate regulatory markers [12]. The lack of consistent correlation between mucosal healing and symptom relief adds further complexity to efficacy assessment, as improvement in one area does not always translate to benefits in the other [62]. In this context, phase 2 trials should prioritize the prevention of histological damage, while phase 3 studies should focus on improving symptoms and enhancing quality of life [139]. In response to these challenges, the U.S. FDA has recommended incorporating validated patient-reported outcomes (PROs) as co-primary endpoints, although disease-specific instruments for CeD are still under development [12,62].

These concerns are reflected in a very recent comprehensive systematic review by AL-Taie et al. The authors concluded that latiglutenase and larazotide acetate currently represent the most advanced candidates for reducing gluten sensitization, while tissue TG2 inhibitors also show potential. However, most data originate from controlled environments that do not adequately reflect real-world variability in gluten exposure. Therefore, it is recommended that future trials should incorporate longer duration or higher dose gluten challenges to more accurately estimate true therapeutic benefits [140]. Similarly, microbiome based interventions are still largely dependent on experimental models, with human trials remaining at an early exploratory stage [141].

### 9.2. Future Perspectives

Despite these barriers, research into CeD continues to advance toward mechanism-based precision therapies. Recent studies have underscored the role of endoplasmic reticulum (ER) stress in the pathogenesis of CeD, suggesting that chemical chaperones may serve as promising therapeutic candidates [142]. Another novel approach involves MTX-101, a bispecific antibody targeting inhibitory killer-cell immunoglobulin-like receptor (KIR) and CD8 receptors, which selectively binds CD8^+^ regulatory T cells without triggering non-specific immune activation or systemic cytokine release [143]. However, translating these preclinical findings into clinical practice remains challenging. Animal models of CeD capture only a limited subset of the disease’s complexity, with significant variations in immune architecture, genetic background, lifespan, and environmental exposure [72,144]. Stem cell-based therapies represent another promising strategy. Mesenchymal stem cells (MSCs) exhibit broad immunomodulatory effects through cell-to-cell interactions and the secretion of bioactive factors, without requiring sustained presence in the intestinal mucosa. While intravenous administration often leads to sequestration of MSCs in the lungs, alternative sources such as umbilical cord, amniotic fluid, and placenta offer distinct advantages. However, critical issues such as optimal dosing, route of administration, and timing still require resolution before these therapies can be effectively translated into clinical use [145,146].

Technological innovations may also transform the preclinical research landscape. HIOs and OOC systems can provide physiologically relevant in vitro models that replicate critical aspects of intestinal structure and function [15,147,148]. Organoids, derived from stem cells, effectively mimic the epithelial architecture and cellular diversity of the gut, while OOC systems simulate the dynamic mechanical and biochemical conditions of the intestinal environment [148]. When co-cultured with immune cells and microbiota, these platforms allow for detailed exploration of epithelial barrier integrity, immune signaling, and gene-environment interactions relevant to gluten-induced inflammation. The integration of microbial communities into CeD-specific OOC models holds particular promise, although accurately replicating the oxygen-sensitive intestinal ecosystem remains a significant technical challenge [15,149].

Computational medicine is another rapidly advancing field with significant implications for CeD. ML technologies are being increasingly applied to enhance diagnostic accuracy and accelerate the discovery of new therapeutic strategies. ML algorithms can identify individuals at high risk for undiagnosed CeD, thereby facilitating targeted screening efforts [150]. Moreover, computer-assisted diagnostic systems may achieve accuracy levels comparable to expert pathologists in distinguishing CeD-positive from CeD-negative biopsies [151]. The integration of clinical, serological, and genomic data through ML is revealing novel molecular networks and genetic determinants of disease susceptibility [152]. Large-scale genomic studies have also uncovered new associations within the HLA class III region, independent of the classical class II loci, thereby expanding the immunogenetic framework of CeD and suggesting potential new therapeutic targets [153,154].

**Figure 4:**
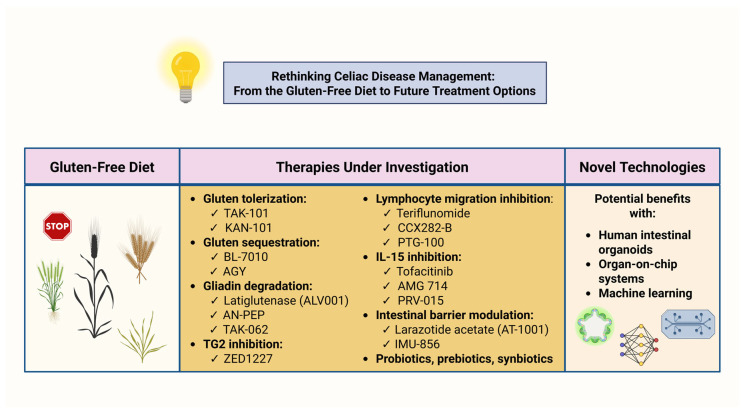
Rethinking celiac disease management: From the gluten-free diet to future treatment options. Abbreviations: AN-PEP: *Aspergillus niger* prolyl endopeptidase; IL-15: interleukin-15; TG2: transglutaminase 2. Created in BioRender. Kounatidis, D. (2025) https://BioRender.com/ctdc9nx (assessed on 18 December 2025).

Collectively, these technological advancements represent a shift from mere descriptive observation to mechanistic precision in CeD research. The key challenge moving forward will be translating these conceptual and technological innovations into clinically effective therapies that alter the disease trajectory and improve long-term outcomes for patients with CeD. Figure 4 summarizes the current landscape of research on emerging therapeutic approaches in CeD.

## 10. Conclusions

The ongoing reliance on strict GFD adherence remains a considerable burden for patients with CeD, highlighting the critical need for more innovative and scalable treatment options. Despite significant advancements in understanding the disease’s pathophysiology and the development of therapeutic agents targeting specific mechanisms, no single therapeutic strategy has yet proven universally effective. While some researchers suggest that the first pharmacological therapy for CeD may become available in the coming years, such a development remains unlikely given the current data, primarily due to the limitations in study design and the lack of long-term safety and efficacy data. In this context, emerging technologies offer promising potential, despite their early-stage development, with the need for better-designed studies being a fundamental prerequisite. Ultimately, the path toward a comprehensive treatment for CeD will depend on sustained collaborative efforts, patient-centered research, and the rigorous execution of well-structured clinical trials.

## Figures and Tables

**Table 1:** Findings from major clinical studies evaluating therapeutic strategies for celiac disease.

Author, Year	Agent Under Investigation	Mechanism of Action	Study Design	Results	Conclusions
Murray, 2022 [78]	Latiglutenase (ALV003)	Gluten degradation	Phase 2, double-blind, placebo-controlled gluten challenge trial of latiglutenase 1200 mg in 43 CeD patients (21 latiglutenase, 22 placebo) receiving 2 g/day gluten for 6 weeks	✓ Latiglutenase largely preserved mucosal structure (Vh:Cd −0.04) compared with greater deterioration in placebo (−0.35), *p* = 0.057 ✓ IEL increase was smaller with latiglutenase (9.8 cells/mm) than placebo (24.8 cells/mm), *p* = 0.018 ✓ Symptom worsening reduced with latiglutenase vs. placebo: abdominal pain 0.22 vs. 1.63, bloating 0.96 vs. 3.29, tiredness 0.02 vs. 3.20; 3 × 2-week trend *p* = 0.014, 0.030, 0.002 ✓ No major safety concerns were observed	Latiglutenase attenuates gluten-induced intestinal mucosal injury and alleviates symptom severity in patients with CeD.
Stefanolo, 2024 [86]	AN-PEP	Gluten degradation	Exploratory, double-blind, randomized, placebo-controlled trial including 40 adults with CeD on a strict GFD. After a 4-week run-in period, participants received AN-PEP or placebo for 4 weeks.	✓ No significant difference in stool GIP levels between AN-PEP and placebo; GIP was undetectable in 65.6% of samples ✓ Median GIP concentration decreased by 44.7% in the AN-PEP arm vs. run-in period ✓ About 30% of patients with detectable GIP during run-in achieved lower or undetectable levels on AN-PEP ✓ Fewer patients reported severe symptoms with AN-PEP (*p* < 0.03) ✓ No significant changes in CeD serology were observed	AN-PEP does not significantly lower overall gluten exposure but is associated with reduced symptom severity, supporting further evaluation of this enzyme as adjunctive therapy for accidental gluten ingestion in CeD.
Pultz, 2021 [87]	TAK-062	Gluten degradation	Phase 1 dose-escalation study evaluating TAK-062 (100–900 mg) in healthy participants and patients with CeD.	✓ In vitro, TAK-062 degraded > 99% of gluten (3–9 g) within 10 min ✓ TAK-062 was well tolerated and achieved from 97% to more than 99% gluten degradation in complex meals containing 1–6 g gluten within 20–65 min post-dose ✓ No safety concerns reported	TAK-062 is safe and effectively degrades large amounts of gluten rapidly, supporting its potential as an oral enzyme therapy for CeD.
Sample, 2017 [89]	AGY	Gluten sequestration	Open-label, single-arm study in 10 adults with biopsy-confirmed CeD on a GFD. After a 2-week run-in, participants received two AGY capsules before meals for 4 weeks.	✓ Most participants reported fewer symptoms, especially tiredness, headache, and bloating ✓ QoL scores improved ✓ Reductions observed in CeD antibodies and LAMA ratio compared with run-in ✓ No safety issues identified	AGY is well-tolerated and shows potential benefits in symptom relief, QoL, and markers of intestinal permeability and immunity.
Schuppan, 2021 [93]	ZED1227	TG2 inhibition	Proof-of-concept, 6-week, randomised, placebo-controlled trial evaluating three doses of ZED1227 (10 mg, 50 mg, 100 mg) vs. placebo in adults with well-controlled CeD undergoing daily gluten challenge: ✓ 10 mg group: 41 patients enrolled, 35 with adequate duodenal-biopsy samples ✓ 50 mg group: 41 patients enrolled, 39 with adequate duodenal-biopsy samples ✓ 100 mg group: 41 patients enrolled, 38 with adequate duodenal-biopsy samples ✓ Placebo group: 40 patients enrolled, 30 with adequate duodenal-biopsy samples	✓ All doses reduced gluten-induced duodenal mucosal injury compared with placebo (Vh:Cd ratio improvement: 0.44–0.49; *p* ≤ 0.001) ✓ Intraepithelial lymphocyte density decreased most with the 100 mg dose (−9.6 cells/100 epithelial cells) ✓ 100 mg dose showed potential improvements in symptoms and QoL scores ✓ Common adverse events were headache, nausea, diarrhea, vomiting, and abdominal pain, while rash occurred in 8% of patients treated with the 100 mg regimen	ZED1227 diminishes gluten-induced duodenal damage in CeD patients, with higher doses showing trends toward greater symptom relief and QoL benefits, and an overall tolerable safety profile.
Kelly, 2021 [94]	TAK-101	Gluten tolerization	Phase 1 dose-escalation and phase 2a double-blind, randomized, placebo-controlled studies of TAK-101 in CeD. In phase 2a, 33 randomized patients completed the 14-day gluten challenge.	✓ TAK-101 reduced gluten-induced IFN-γ spot-forming units by 88% vs. placebo (2.01 vs. 17.58; *p* = 0.006) ✓ Vh:Cd ration declined in the placebo group (−0.63; *p* = 0.002) but remained stable in the TAK-101 group (−0.18; *p* = 0.110); the intergroup difference was not statistically significant (*p* = 0.08) ✓ IEL counts were unchanged ✓ TAK-101 reduced circulating α4β7^+^CD4^+^, αEβ7^+^CD8^+^, and γδ effector memory T cells (all *p* < 0.05) ✓ No serious adverse events were reported	TAK-101 is safe, and well tolerated, and effectively prevents gluten-induced immune activation in subjects with CeD.
Murray, 2023 [95]	KAN-101	Gluten tolerization	Phase 1 study of KAN-101 in adults with *HLA-DQ2.5*-positive CeD: ✓ Part A (14 patients): open-label, single ascending intravenous doses (0.15–1.5 mg/kg) ✓ Part B (27 patients): randomized, placebo-controlled, multiple ascending doses (0.15–0.6 mg/kg) with a subsequent 3-day gluten challenge	✓ Treatment-related AEs: 79% in part A, 76% in KAN-101 part B, mostly mild to moderate (nausea, diarrhea, abdominal pain, vomiting) ✓ No serious AEs or deaths ✓ KAN-101 cleared rapidly (half-life 3.7–31.7 min), with no accumulation on repeated dosing	KAN-101 is well tolerated with no dose-limiting toxicities, demonstrating rapid systemic clearance and acceptable safety.
Risnes, 2025 [97]	Teriflunomide	Lymphocyte migration inhibition	Phase 2a RCT in 15 patients with CeD, comparing teriflunomide vs. placebo during a 3-day oral gluten challenge after a loading dose.	No significant difference observed in gluten-specific T cell activation markers between teriflunomide and placebo group.	Teriflunomide is not effective as a non-dietary therapeutic option for patients with CeD
Dieckman, 2024 [103]	Tofacitinib	IL-15 inhibition	Open-label, prospective pilot study evaluating tofacitinib (10 mg twice daily for 12 weeks) in 6 patients with therapy-RCDII, including 4 per-protocol and 2 off-protocol participants.	✓ All patients completed treatment ✓ Per-protocol patients showed rapid symptom resolution within 2–14 days and significant weight gain (median +12.3% at week 12) ✓ Symptoms recurred after discontinuation but resolved promptly upon restarting therapy ✓ Histologic improvement of villous atrophy observed in 4 of 6 patients; marked mucosal healing in ulcerative jejunitis cases ✓ No reduction in the proportion of aberrant IELs (primary endpoint unmet) Most common AE: transient lymphopenia; one pulmonary embolism during catheter sepsis. No opportunistic infections or lymphoma observed ✓ Extended follow-up (median 84 weeks) showed sustained clinical and histologic remission with continued tofacitinib	Tofacitinib may represent a promising targeted therapy for RCDII and warrants further investigation.
Paterson, 2007 [107]	AT-1001	Intestinal barrier modulation	In-patient, double-blind, randomized, placebo-controlled safety study, involving 24 subjects	✓ Placebo group showed a 70% rise in intestinal permeability after gluten challenge, whereas AT-1001 maintained normal permeability ✓ IFN-γ elevation occurred in 57% of placebo subjects vs. 29% in those receiving AT-1001 ✓ AT-1001 did not increase adverse events compared with placebo ✓ Gastrointestinal symptoms were notably more common in the placebo arm (*p* = 0.018)	AT-1001 is safe and well tolerated, demonstrating potential to preserve intestinal integrity, limit inflammatory cytokine activation, and lessen gastrointestinal discomfort in CeD patients following gluten exposure.
Daveson, 2025 [112]	IMU-856	Intestinal barrier modulation	Three-part, double-blind, randomized, placebo-controlled, phase 1 trial: ✓ Part A: 33 healthy participants received single ascending IMU-856 doses (10–160 mg) or placebo (3:1 randomization) ✓ Part B: 19 healthy participants received once-daily IMU-856 for 14 days or placebo (3:1 randomization) ✓ Part C: 43 patients with well-controlled CeD randomized 1:1:1 to IMU-856 80 mg (*n* = 14), IMU-856 160 mg (*n* = 15), or placebo (*n* = 14) ✓ Part C included a 28-day dosing period with a 15-day gluten challenge starting on day 14	✓ No dose-limiting toxicities or deaths were observed across single and multiple dose cohorts ✓ TEAEs were mostly mild and occurred at similar frequency to placebo across all study parts ✓ In Part C, gastrointestinal TEAEs (nausea, diarrhea, abdominal distension) were more common with IMU-856 but were predominantly mild and self-limiting ✓ Two serious adverse events occurred (bacterial myocarditis, biliary colic), both assessed as unrelated to IMU-856 ✓ IMU-856 groups showed substantially less Vh reduction during gluten challenge (−21 to −23 μm) compared with placebo (−60 μm), indicating attenuation of gluten-induced mucosal damage	IMU-856 is safe and generally well tolerated in patients with CeD, supporting its further evaluation in future clinical trials.

Abbreviations: AE: adverse event; AN-PEP: Aspergillus niger prolyl endopeptidase; CeD: Celiac disease; GIP: gliadin immunogenic peptides; GFD: gluten-free diet; IFN-γ: interferon-gamma; IEL: intraepithelial lymphocyte; IL-15: interleukin-15; LAMA: lactulose-to-mannitol; QoL: quality of life; RCDII: refractory celiac disease type 2; RCT: randomized clinical trial; TEAE: treatment-emergent adverse event; Vh:Cd: villus height-to-crypt depth ratio.

**Table 2:** Main ongoing clinical studies exploring therapeutic strategies for celiac disease.

Clinical Trial	Agent	Mechanism of Action	Study Design	Study Objective	Study Status
NCT04839575	Latiglutenase (ALV003)	Gluten degradation	Phase 2, single-center, prospective, double-blind, placebo-controlled, crossover clinical trial.	To evaluate the efficacy and safety of latiglutenase in participants with T1D and CeD who are on a GFD and undergoing periodic gluten exposure.	Terminated (due to COVID-19 interruptions and enrollment difficulties)
NCT04243551	Latiglutenase	Gluten degradation	Phase 2b, multicenter, prospective, randomized, double-blind, placebo-controlled, crossover clinical trial.	To explore the efficacy and safety of latiglutenase in symptomatic CeD patients on a GFD who are undergoing periodic gluten exposure.	Terminated (due to low enrollment related to COVID-19)
NCT01917630	Latiglutenase	Gluten degradation	Phase 2b, randomized, double-blind, placebo-controlled, dose-ranging clinical trial.	To assess the effects of 12-week administration of latiglutenase at different dose levels on small intestinal mucosa and CeD symptoms in patients maintaining a GFD.	Unknown
NCT04788797	AN-PEP	Gluten degradation	Interventional, prospective, randomized, comparative, double-blind clinical trial.	To evaluate the effect of daily AN-PEP administration vs. placebo on GIP excretion in stool and urine in CeD patients, as a measure of gluten detoxification under real-life dietary conditions.	Completed
NCT05353985	TAK-062	Gluten degradation	Phase 2, multicenter, randomized, double-blind, placebo-controlled, dose-ranging clinical trial.	To evaluate the efficacy and safety of TAK-062 in reducing CeD-related symptoms and improving intestinal damage from gluten exposure in participants with active CeD attempting to maintain a GFD.	Completed
NCT01990885	BL-7010	Gluten sequestration	Two-part, randomized, double-blind, placebo-controlled clinical trial.	To assess the safety of single and repeated oral doses of BL-7010 in well-controlled patients with CeD and to determine whether the compound is systemically absorbed.	Completed
NCT03707730	AGY	Gluten sequestration	Randomized, double-blind, placebo-controlled, crossover clinical trial.	To evaluate the efficacy and safety of AGY compared to placebo in individuals aged 10–65 with confirmed CeD on a GFD, focusing on symptom improvement, QoL, autoantibodies, and gut permeability.	Completed
NCT05818956	TAK-227	TG2 inhibition	Randomized, open-label, single-dose, three-way crossover clinical trial in healthy adults.	To evaluate the effect of food on the pharmacokinetics, safety, and tolerability of a single 50 mg dose of TAK-227 and to determine optimal dosing conditions.	Completed
NCT04530123	TAK-101	Gluten tolerization	Randomized, double-blind, placebo-controlled, phase 2, dose-ranging clinical trial.	To evaluate whether TAK-101 can reduce gluten-related symptoms and immune activation in adults with celiac disease on a gluten-free diet, while assessing its safety and efficacy across different dose levels.	Active, not recruiting
NCT06001177	KAN-101	Gluten tolerization	Multi-center, double-blind, placebo-controlled, phase 2a clinical trial.	To investigate the efficacy, safety, and tolerability of KAN-101 in adults with CeD on a GFD, specifically evaluating its ability to protect against gluten-induced histological changes in the duodenum.	Completed
NCT00540657	CCX282-B	Lymphocyte migration inhibition	Randomized, double-blind, placebo-controlled, phase 2 clinical trial.	To investigate the efficacy of CCX282-B in reducing the effects of gluten ingestion in patients with CeD.	Completed
NCT04524221	PTG-100	Lymphocyte migration inhibition	Phase 1b, randomized, placebo-controlled clinical trial	To evaluate the safety and efficacy of PTG-100 in preventing gluten-induced inflammatory injury to the small intestine in patients with CeD	Completed
NCT02633020	AMG 714	IL-15 inhibition	Phase 2a, randomized, double-blind, placebo-controlled, parallel-group clinical trial	To assess the efficacy and safety of AMG 714 in adult patients with type II refractory CeD	Completed
NCT04424927	PRV-015	IL-15 inhibition	Phase 2b, randomized, double-blind, placebo-controlled, parallel-group clinical trial	To evaluate the efficacy and safety of three dose regimens of PRV-015 in adult patients with non-responsive CeD who continue to experience symptoms despite adhering to a GFD	Completed
NCT03569007	Larazotide acetate (AT-1001)	Intestinal barrier modulation	Phase 3, randomized, double-blind, placebo-controlled, multicenter clinical trial.	To assess the efficacy and safety of larazotide acetate in relieving persistent symptoms in adult CeD patients on a GFD.	Terminated (by sponsor)

Abbreviations: AN-PEP: *Aspergillus niger* prolyl endopeptidase; CeD: celiac disease; GFD: gluten-free diet; GIP: gliadin immunogenic peptides; IL-15: interleukin-15; T1D: type 1 diabetes; TG2: transglutaminase 2; QoL: quality of life.

## Data Availability

No new data were created or analyzed in this study. Data sharing is not applicable to this article.

## Abbreviations

AE: adverse event; APC: antigen-presenting cell; AN-PEP: *Aspergillus niger* prolyl endopeptidase; BCAA: branched-chain amino acid; Breg: regulatory B cell; CCL25: CC chemokine ligand 25; CCR9: CC chemokine receptor 9; COX: cyclooxygenase; CRA: cytokine release assay; DDE: p,p′-dichlorodiphenyldichloroethylene; DGP: deamidated gliadin peptides; EATL: enteropathy-associated T-cell lymphoma; ER: endoplasmic reticulum; FDA: Food and Drug Administration; FMT: fecal microbiota transplantation; FOXP3: forkhead box P3; GWAS: genome-wide association studies; GIP: gliadin immunogenic peptides; HERVs: human endogenous retroviruses; HIOs: human intestinal organoids; HLA: human leukocyte antigen; IFN-γ: interferon-gamma; IgA: immunoglobulin A; IgG: immunoglobulin G; IEL: intraepithelial lymphocyte; JAK: Janus kinase; JAM: junctional adhesion molecule; KIR: killer-cell immunoglobulin-like receptor; LAMA: lactulose-to-mannitol; LINE-1: long interspersed nuclear elements 1; lncRNAs: long non-coding RNAs; LPS: lipopolysaccharide; ML: machine learning; MAdCAM-1: mucosal addressin cell adhesion molecule 1; MSC: mesenchymal stem cell; NF-κB: nuclear factor kappa-light-chain-enhancer of activated B cells; NK: natural killer; NKG2D: natural killer group 2D; POP: persistent organic pollutant; PROs: patient-reported outcomes; RBC: red blood cell; RCDII: refractory CeD type II; RCT: randomized clinical trial; RV: rotavirus; SCFA: short-chain fatty acid; sIgAD: selective IgA deficiency; SIRT6: sirtuin-6; SNP: single-nucleotide polymorphism; SETDB1: SET domain bifurcated histone lysine methyltransferase 1; TEAE: treatment-emergent adverse event; TG: transglutaminase; TLR: Toll-like receptor; TNF-α: tumor necrosis factor-alpha; Treg: regulatory T cell; TRIM28: tripartite motif containing 28; Vh:Cd: villus height to crypt depth; QoL: quality of life.
